# Non-tuberculous mycobacterial infective endocarditis in a tuberculosis-endemic region after recent cardiovascular procedures: a case series

**DOI:** 10.1099/acmi.0.001130.v3

**Published:** 2026-02-19

**Authors:** Shohael Mahmud Arafat, Chowdhury Adnan Sami, Abed Hussain Khan, Sudip Kumar Banik, Md. Mizanur Rahman Khan, Lovely Barai

**Affiliations:** 1 Department of Internal Medicine, Bangabandhu Sheikh Mujib Medical University, Dhaka, Bangladesh; 2 Department of Microbiology, Bangladesh Institute of Research and Rehabilitation of Diabetes, Endocrine and Metabolic Disorders, Dhaka, Bangladesh

**Keywords:** culture-negative endocarditis, infective endocarditis (IE), non-tuberculous mycobacteria (NTM), percutaneous coronary intervention (PCI), rapid-growing mycobacteria

## Abstract

**Background.** Non-tuberculous mycobacterial infective endocarditis (NTM-IE) is an uncommon but increasingly recognized aetiology of culture-negative endocarditis, particularly in the context of healthcare exposure. Rapidly growing non-tuberculous mycobacteria (NTM) species present significant diagnostic and therapeutic challenges due to their indolent nature and clinical similarity to tuberculosis.

**Case summary.** We describe a case series of three patients with native valve infective endocarditis caused by rapidly growing NTM following recent percutaneous coronary intervention. All patients initially presented with prolonged fever and systemic inflammatory signs, and routine microbiological workup results were negative. The diagnosis was based on repeated blood and/or urine cultures with the detection of rapidly growing NTM, exclusion of *Mycobacterium tuberculosis* by PCR analysis and echocardiography demonstrating valvular vegetations. Cultures were performed on consecutive days at a single reference laboratory according to established protocols to reduce the risk of sample contamination. Species-level identification was not feasible because of limited resources. All patients received combination antimicrobial therapy guided by the available susceptibility data and expert consultation. Despite multidrug treatment, clinical outcomes were poor in these cases. Two patients died before definitive surgical intervention could be performed, and one patient died during the induction of the valve replacement surgery.

**Conclusion.** This case series highlights the difficulties in diagnosing NTM-IE and its high mortality rate. NTM infection should be considered in patients with chronic fever after invasive cardiovascular procedures or with prolonged culture-negative endocarditis. Medical therapy is frequently inadequate, and a combined medical–surgical approach may be necessary.

## Data Summary

No data, tools, software or code were generated or required for our work.

## Introduction

Non-tuberculous mycobacterial infective endocarditis (NTM-IE) after cardiovascular procedures, especially post-percutaneous coronary intervention (PCI), is a less frequent but serious complication [1]. NTM-IE is frequently associated with rapidly growing mycobacteria, such as *Mycobacterium fortuitum* and *Mycobacterium abscessus*, which are resistant to common treatments and are responsible for culture-negative endocarditis [2]. The clinical picture may be difficult to determine because of the insidious nature of the infection and the scarcity of blood culture results. Diagnosis is challenging because routine cultures are often negative, and the clinical presentation overlaps with that of tuberculosis [2]. Treatment of NTM-IE includes surgical and medical approaches, and in some cases, complicated regimens of antibiotics and valve replacement are required [2].

We present three cases of non-tuberculous mycobacteria (NTM)-associated native valve infective endocarditis (IE) that developed following coronary stenting.

## Methods

### Microbiological identification and susceptibility testing

All microbiological investigations for all three cases were performed at the Department of Microbiology, Bangladesh Institute of Research and Rehabilitation in Diabetes, Endocrine, and Metabolic Disorders (BIRDEM). The same standardized laboratory protocols and quality control procedures were uniformly applied across all cases.

The isolates recovered from blood and/or urine cultures were phenotypically identified as rapidly growing NTM based on a combination of microbiological criteria, including growth within 7 days, characteristic colony morphology, Ziehl–Neelsen positivity, Gram-stain appearance from culture growth and absence of pigmentation on subculture in Lowenstein–Jensen medium. In addition, real-time PCR was performed on DNA extracted from cultured colonies using a commercial assay capable of differentiating the *Mycobacterium tuberculosis* complex from NTM (Viasure Real-Time PCR Detection Kit; Certest Biotech, Zaragoza, Spain), thereby excluding *M. tuberculosis* as the causative organism [3].

Species-level identification could not be performed because of the unavailability of MALDI-TOF or gene sequencing facilities at our centre, which we acknowledge as a limitation. Antimicrobial susceptibility testing was performed using the broth microdilution method with cation-adjusted Mueller–Hinton broth, in accordance with Clinical and Laboratory Standards Institute (CLSI M24) (third edition) guidelines [4].

To exclude laboratory contamination, repeat blood and/or urine cultures were done over three successive days in each case, all of which yielded concordant growth of rapidly growing NTM.

## Case summary

### Case 1

#### Clinical presentation

A 63-year-old man with long-standing diabetes mellitus and hypertension underwent PCI for double-vessel coronary artery disease via the right radial artery. Two months later, he started having intermittent high-grade fever, which went up to 104 °F and persisted for 6 months, along with a weight loss of 6 kg within the same period. There was no history of tuberculosis or known contact with patient having tuberculosis.

#### Diagnostic work-up

Initial testing for infectious causes was performed. Urine microscopy revealed acid-fast bacilli (AFB) on Ziehl–Neelsen staining, and the interferon-gamma release assay (QuantiFERON-TB Gold) was positive, prompting suspicion of genitourinary tuberculosis. The GeneXpert MTB/RIF test and routine urine cultures were negative. The patient did not improve clinically despite the initiation of empirical first-line antitubercular therapy.

**Fig. 1.**
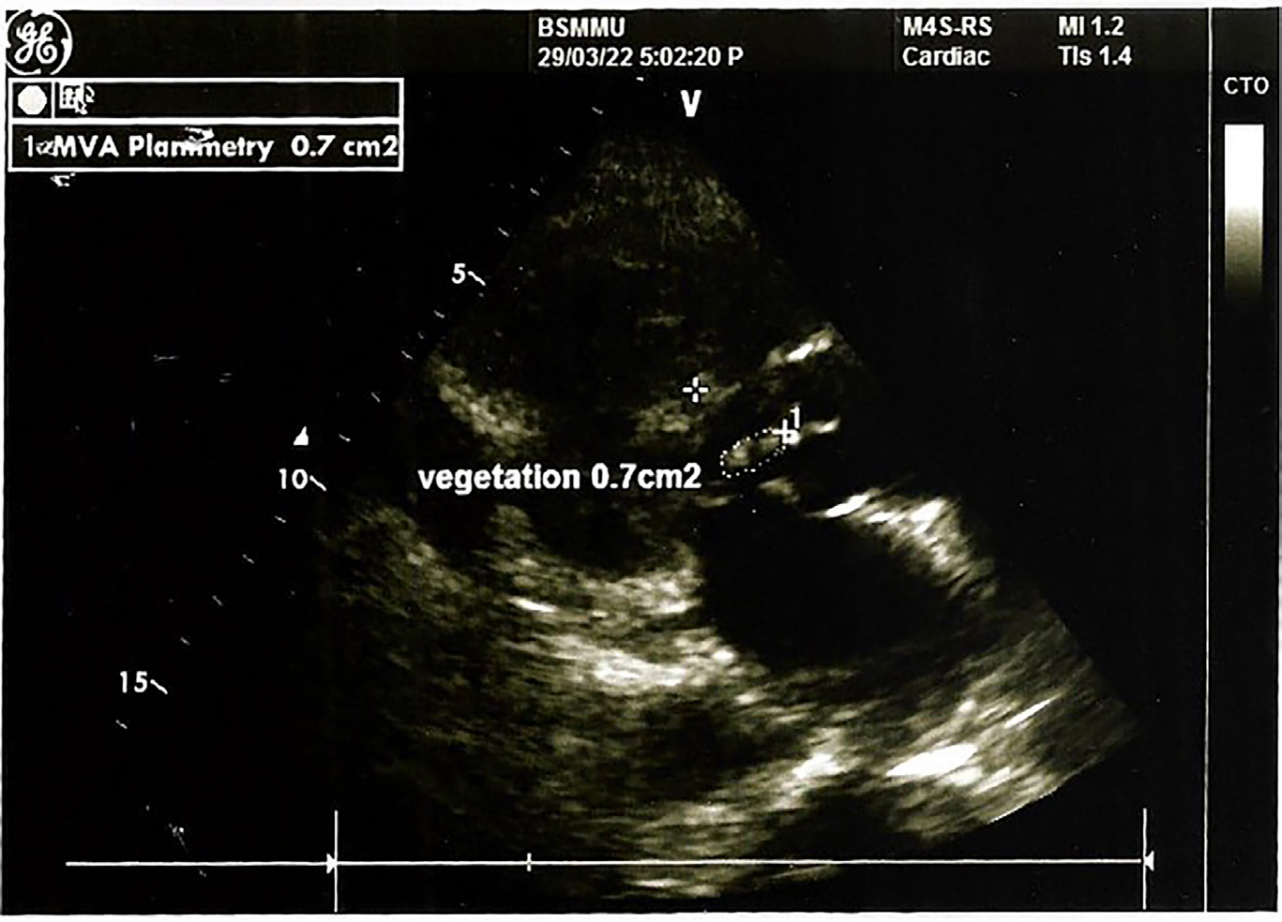
Vegetationat the tip of the anterior mitralleaflet.

**Fig. 2.**
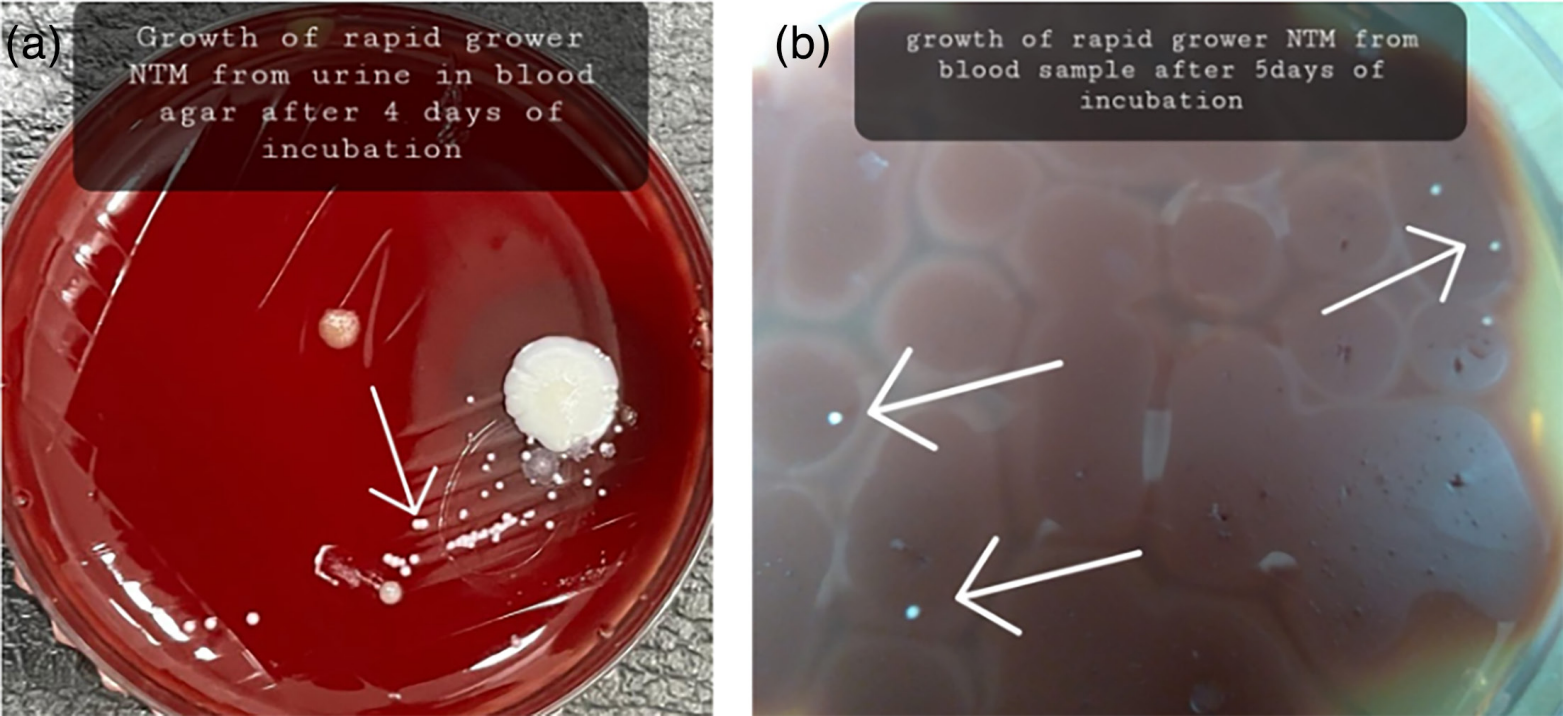
Rapid grower nontuberculous mycobacterium (NTM) in (a) urine, and in (b)blood.

Given the persistent fever despite appropriate antituberculosis treatment, alternative diagnoses were considered. Transthoracic echocardiography was performed to evaluate IE and revealed a small vegetation on the anterior mitral leaflet (Fig. 1). Prolonged blood and urine cultures subsequently yielded rapidly growing NTM after 5 days of incubation (Fig. 2a, b). The relevant laboratory findings are presented in Table 1.

**Table 1.** Investigation profile of all three patients

Investigation	Case 1	Case 2	Case 3
Haemoglobin (g dl^−1^)	9.5	8.3	7.4
ESR (mm in first hour)	120	45	06
WBC×10^9^ per litre	7	4.5	3.5
Platelets×10^9^ per litre	300	110	105
C-reactive protein (mg dl^−1^)	39.38	72.37	127.18
Peripheral blood film	Dimorphic anaemia	Pancytopenia	Microcytic hypochromic anaemia
Creatinine (mg dl^−1^)	1.11	3.9	1.28
ALT (U l^−1^)	54	39	42
CXR P/A view	Normal	Normal	Normal
USG of whole abdomen	Normal	Normal	Hepatosplenomegaly
Transoesophageal echocardiography	Small vegetation at the tip of anterior mitral leaflet	Vegetation right coronary cusp (7×3 mm) and NCC (5×5 mm)	Vegetation attached to NCC of aortic valve (07×03 mm)
Blood culture	Rapid grower NTM (after 5 days of incubation)	Rapid grower NTM (after 4 days of incubation)	Rapid grower NTM (after 4 days of incubation)
Urine microscopy (Z–N stain)	AFB present	Not present	Not present
Urine culture (in blood agar)	Rapid grower NTM (after 4 days of incubation)	No growth	No growth
Bone marrow	Normal	Normal	Normal
NT proBNP (pg ml^−1^)	2,100	14,977	510
ICT for malaria and kala-azar	Negative	Negative	Negative
Anti-leptospira IgM and IgG	Negative	Negative	Negative
Anti-HIV (1+2)	Negative	Negative	Negative
ANA	Negative	Negative	Negative

ALT, alanine aminotransferase; ANA, Antinuclear antibody; CXR P/A, chest X-ray (posteroanterior view); HIV, human immunodeficiency virus; ICT, immunochromatographic test (rapid test); NCC, non-coronary cusp; NT-proBNP, N-terminal pro–B-type natriuretic peptide; USG, ultrasonography; WBC, white blood cell count; Z–N, Ziehl–Neelsen stain.

#### Differential diagnosis

Based on the clinical presentation and initial investigations, the differential diagnoses included culture-negative IE, disseminated NTM infection, renal tuberculosis and fungal endocarditis. The lack of response to antituberculosis therapy, echocardiographic evidence of valvular involvement and repeated isolation of rapidly growing NTM supported the diagnosis of NTM-IE.

#### Management

Following microbiological confirmation, combination antimicrobial therapy was initiated with intravenous amikacin, oral clarithromycin and oral linezolid, and subsequently adjusted in consultation with infectious disease and clinical microbiology specialists, guided by the available culture and susceptibility results. The patient became afebrile after 2 weeks of treatment. Amikacin dosing was subsequently adjusted because of rising serum creatinine levels (peak value, 2.02 mg dl^−1^). After 5 weeks of inpatient treatment, the patient was discharged against medical advice (DAMA) with instructions for close outpatient follow-up and renal function monitoring.

#### Outcome

Six weeks after discharge, the patient presented with acute-onset chest pain and dyspnoea. Serum troponin I levels were markedly elevated (4,110.25 ng ml^−1^), and electrocardiography showed nonspecific ST-segment changes. He was managed for a non-ST-segment elevation myocardial infarction but deteriorated rapidly and died within 13 h of admission to the coronary care unit. Adherence to antimicrobial therapy during the interim period following discharge could not be determined reliably.

### Case 2

#### Clinical presentation

A 55-year-old woman with long-standing diabetes mellitus and hypertension presented with a 1-month history of intermittent high-grade fever. She had undergone PCI through the right femoral artery for triple-vessel coronary artery disease 2 months before symptom onset. On admission, she was febrile, pale, tachycardic and tachypnoeic, with bilateral basal crepitations on chest auscultation.

#### Diagnostic work-up

**Fig. 3.**
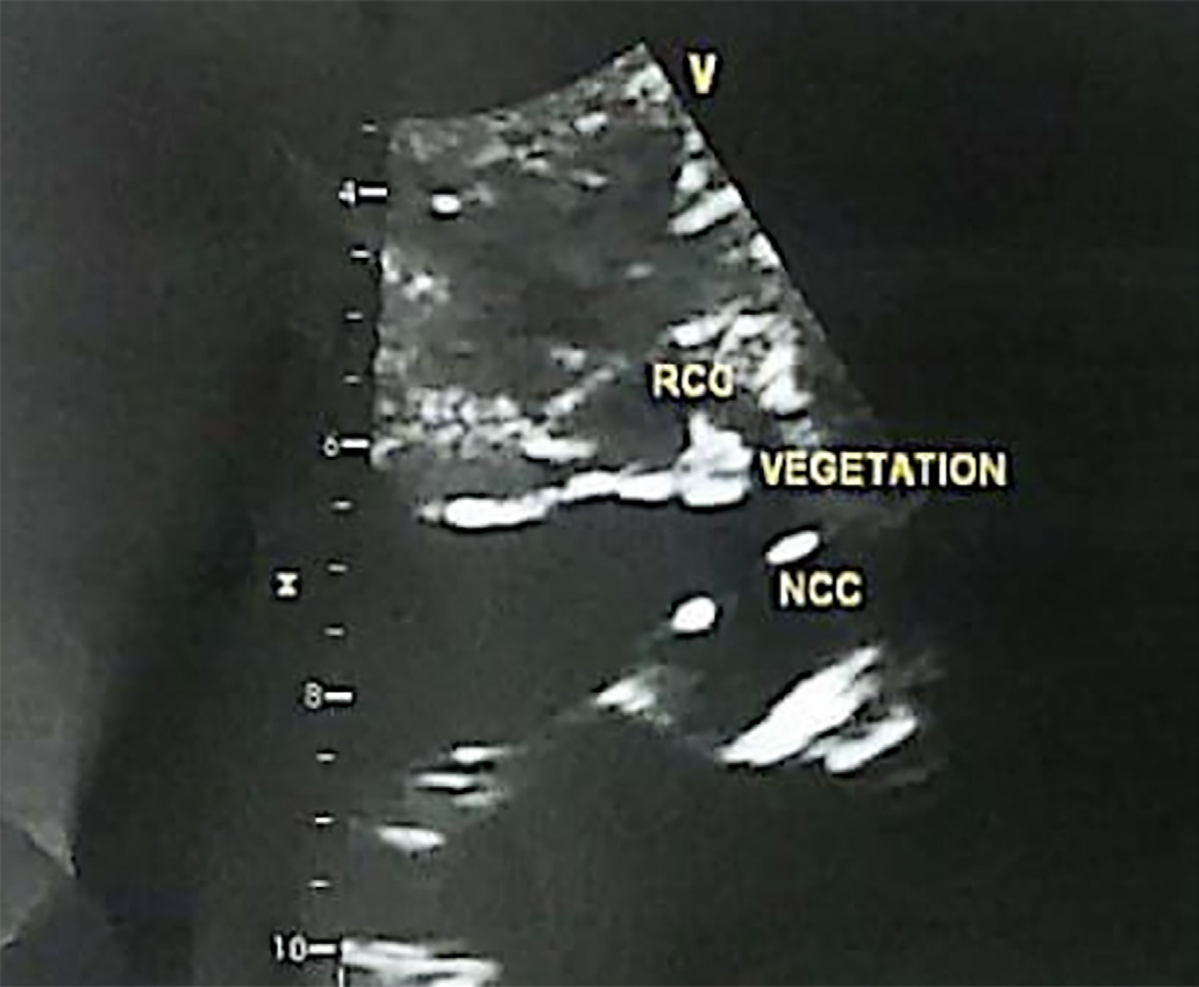
Vegetation of non-coronary cuspNCC of aortic valve. NCC, non-coronarycusp.

The initial laboratory work-up demonstrated pancytopenia and significantly elevated inflammatory markers (Table 1). An extensive evaluation for infectious and autoimmune aetiologies was performed in view of the persistent fever in a patient with recent healthcare exposure, and all initial investigations were negative. Transthoracic echocardiography was used to screen for IE and showed vegetations on the right and non-coronary cusps of the aortic valve (Fig. 3).

**Fig. 4.**
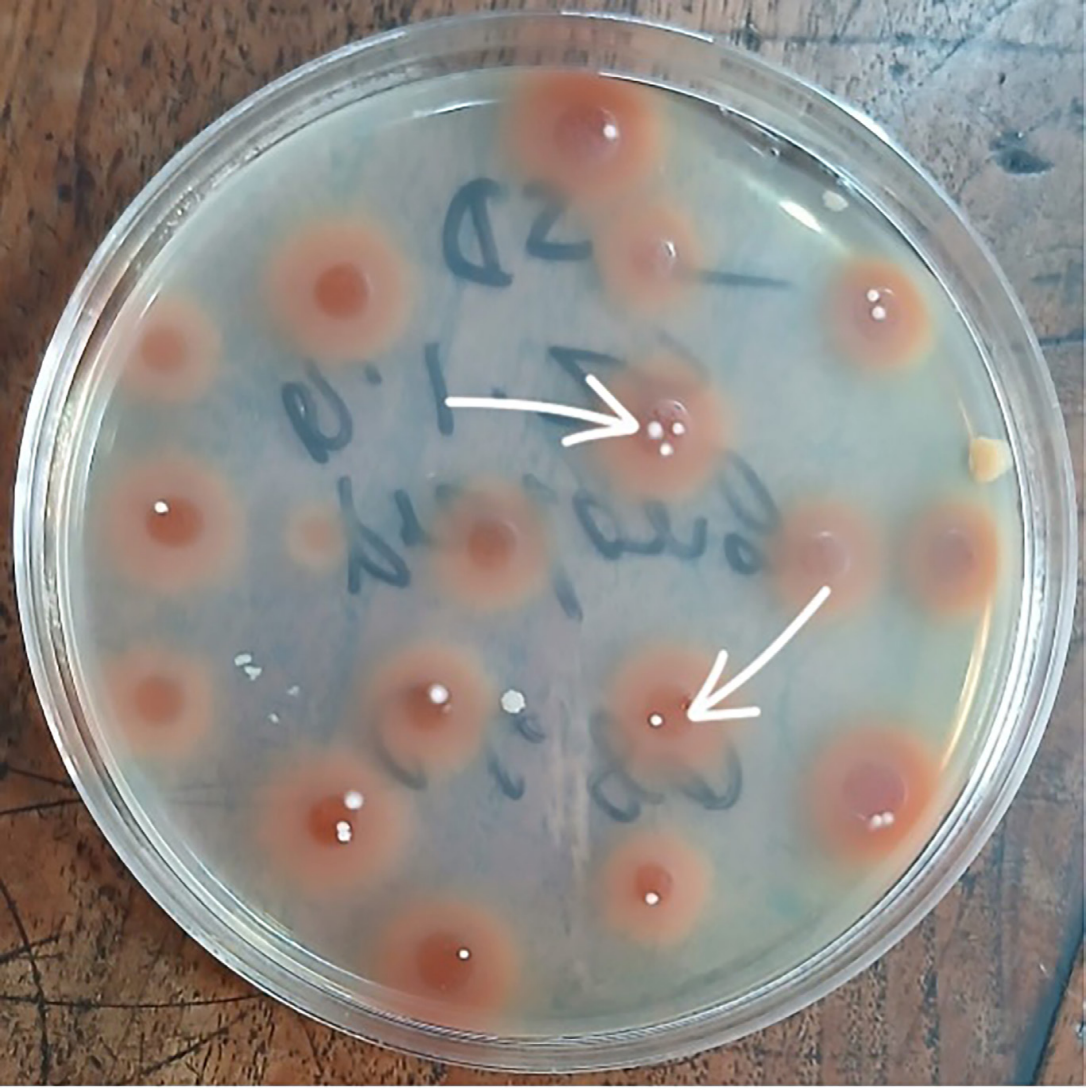
Sabouraud dextrose agar showing rapidly growing NTM

Given the echocardiographic appearance and persistent systemic inflammation, prolonged blood cultures were collected and ultimately grew rapidly growing NTM after extended incubation (Fig. 4), using the standard microbiology preparation described above. At this juncture, the differential diagnoses included culture-negative IE, tuberculous endocarditis, disseminated NTM infection and fungal endocarditis. The recovery of fast-growing NTM from blood cultures, along with valvular vegetations, was consistent with the diagnosis of NTM endocarditis.

#### Management

The patient was started on combination therapy with linezolid, clarithromycin, amikacin and ceftazidime–avibactam. This choice was based on cultural sensitivity and the context of severe sepsis, with the advice of an infectious disease expert. The patient was initially afebrile; however, linezolid was discontinued because of worsening thrombocytopenia, and fever reappeared. Surgical treatment was recommended because of persistent infection despite the use of multiple drugs and echocardiographic findings suggesting active aortic valve disease. Owing to logistic constraints for valve replacement surgery at that time, the patient was referred outside the country.

#### Outcome

She was haemodynamically stable, with no further deterioration of clinical parameters during transfer until surgery. However, she experienced an unexpected cardiac arrest while undergoing anaesthesia induction for elective aortic valve replacement. She was eventually declared dead in the operating theatre despite aggressive resuscitation efforts.

### Case 3

#### Clinical presentation

A 45-year-old man with hypertension, a college teacher from Barishal, developed an acute myocardial infarction (AMI) and was treated with PCI through the right femoral artery for AMI. He presented with a persistent high-grade fever ~1 month after PCI, which persisted for 5 months before consultation. He reported no weight loss, night sweats, cough or other constitutional symptoms that were consistent with tuberculosis. There was no history of tuberculosis or known contact with any patients with tuberculosis.

#### Diagnostic work-up

**Fig. 5.**
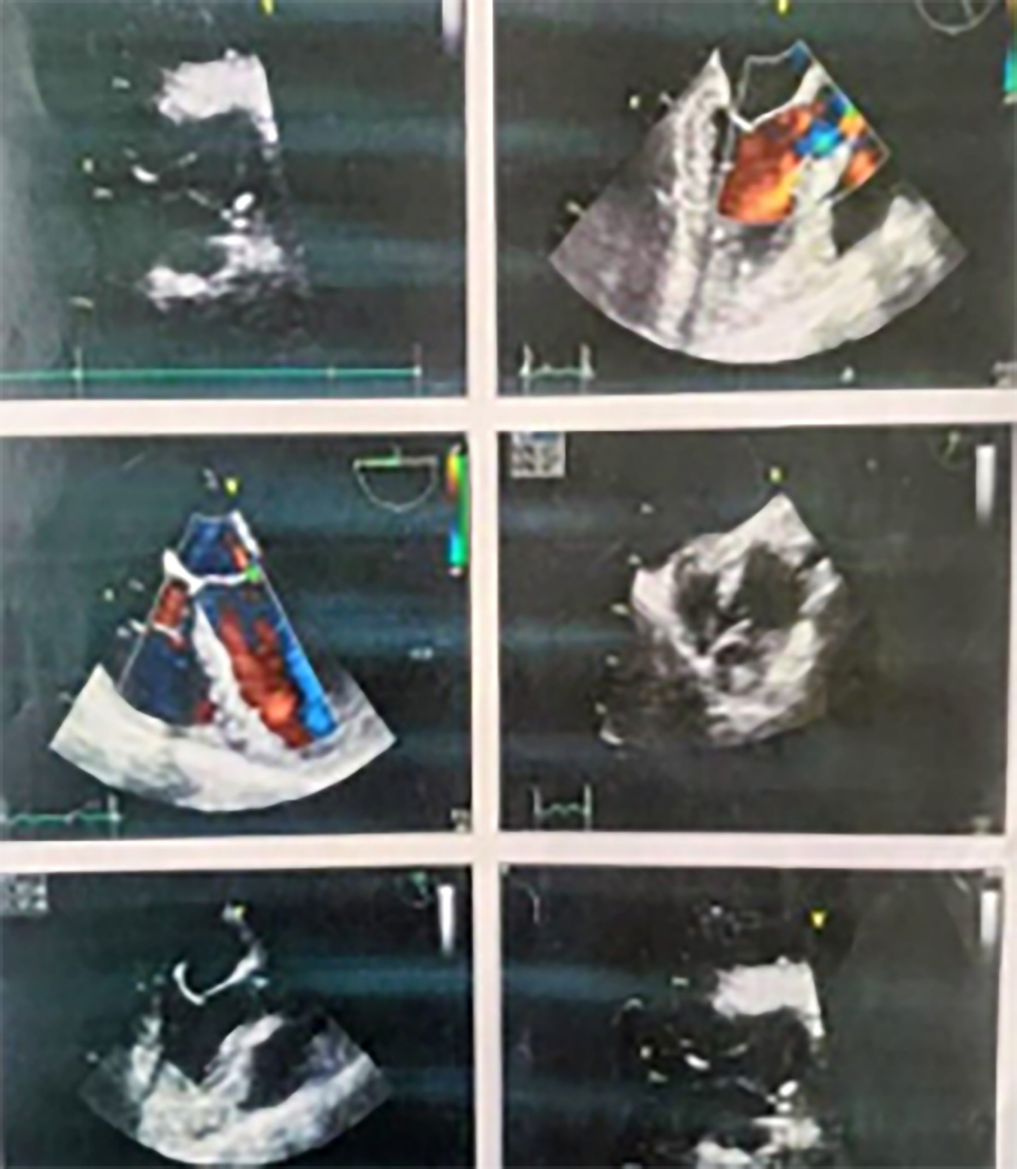
Vegetation attached to non-coronary cusp (NCC) of aortic valve. NCC, non-coronary cusp.

Clinically, the patient appeared pale and febrile. He had splinter haemorrhages on general examination and hepatosplenomegaly on abdominal examination. Laboratory studies were consistent with anaemia, leucopenia and a marked elevation of C-reactive protein levels (Table 1). Due to persistent fever, along with peripheral signs of endocarditis and a history of cardiac procedures, transthoracic echocardiography was performed, which revealed a vegetation attached to the non-coronary cusp of the aortic valve (Fig. 5).

#### Differential diagnosis

**Fig. 6.**
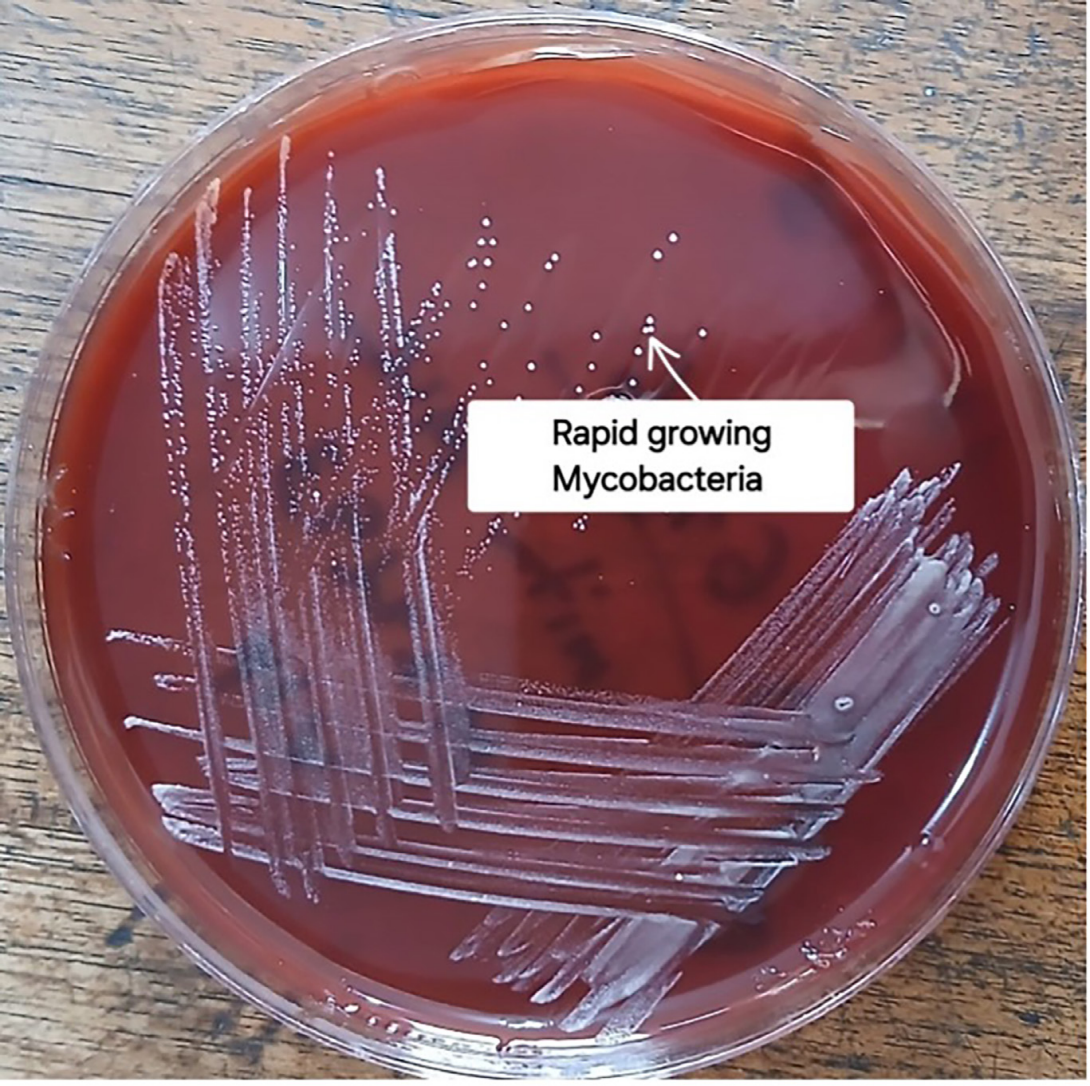
Blood agar media showing rapidly growing NTM.

A comprehensive work-up for infectious and autoimmune aetiologies was performed. Tests for tuberculosis, viral infections, connective tissue disease and haematologic malignancy were all negative. Blood cultures were processed using standardized microbiology procedures, as described earlier, and repeatedly produced rapidly growing NTM (Fig. 6).

Based on the clinical contexts and investigations, culture-negative IE, NTM infection, tuberculosis and haematological malignancy were part of the differential diagnoses. Valvular vegetation, recurrent isolation of rapidly growing NTM from blood cultures and the exclusion of other diagnoses confirmed the diagnosis of NTM-IE involving the native aortic valve.

#### Management

The patient was started on combination antimicrobial therapy involving amikacin, macrolide and linezolid based on the culture sensitivity of the organism, clinical severity, tolerability and expert opinion. Treatment was complicated by cytopenias and the need for close monitoring of renal function and haematological parameters. Despite antimicrobial therapy, the patient showed progressive clinical deterioration. Surgical intervention was considered; however, definitive surgical planning could not be undertaken because of the advanced disease and rapid clinical decline.

#### Outcome

The patient continued to deteriorate, with features of progressive IE and a systemic inflammatory response. The patient was DAMA because he was unable to bear the hospital expenses due to financial constraints. During the second month of treatment, he collapsed at home and was brought to a local hospital, where he was declared dead. The cause of death was most likely cardiac decompensation secondary to advanced NTM-related aortic valve IE.

## Discussion

### Changing epidemiology and risk factors

NTM-IE remains a rare diagnosis but is being reported with increasing frequency owing to the widespread use of intravascular devices and cardiovascular interventions. In the largest systematic review to date (167 patients), rapidly growing species accounted for nearly all native valve infections, and the overall mortality approached 45% [2]. Conservative management without surgery was associated with far poorer survival than combined medical–surgical therapy (mortality, 66.7% versus 30.6%) [2], underscoring the seriousness of these infections. Prosthetic valves were the most common risk factor in that review (63.5% of cases) [2]; however, native valve infections, such as those in our series, have also been linked to rapidly growing mycobacteria. NTM are ubiquitous environmental bacteria. They are classified into rapid-growing species (growth within 7–14 days) and slow-growing species (growth after 14 days) [2,5]. Outbreak investigations have implicated contaminated water sources, heater-cooler devices, hydrotherapy equipment, intravenous infusions and other invasive procedures as vehicles for transmission [5,6]. Our three patients developed fever within weeks to months of PCI; although a causal relationship cannot be established, these procedures represent plausible healthcare-associated exposures.

### Diagnostic challenges and microbiological considerations

#### Blood culture negativity and missed diagnosis

NTM-IE frequently presents as blood culture-negative IE (BCNE). BCNE is defined as endocarditis in which three or more aerobic and anaerobic blood cultures remain negative despite prolonged incubation beyond 1 week [7]. Routine blood cultures may be falsely negative in NTM-IE because rapidly growing species can take 7–10 days to appear, and slow-growing species require more than 2 weeks [2]. In our first case, an AFB smear of urine suggested renal tuberculosis, and the patient received standard antitubercular therapy. AFB smears cannot differentiate the *M. tuberculosis* complex from NTM, and other organisms may stain acid-fast [8]. Only prolonged mycobacterial cultures can detect small numbers of bacilli (up to 8 weeks of incubation), and species-level identification usually requires technologies such as MALDI-TOF mass spectrometry [9]. In the absence of these resources, clinicians should maintain a high index of suspicion for NTM when culture-negative endocarditis follows a recent invasive procedure or when acid-fast organisms are observed.

#### Contamination control and laboratory reproducibility

Because NTM are ubiquitous in water and soil, repeated isolation is essential to distinguish between infection and contamination. Each of our patients had blood and/or urine cultures repeated on consecutive days; the cultures grew the same rapidly growing NTM, and the isolates were processed at a single reference laboratory using standard protocols, thus minimizing the likelihood of contamination. These findings meet the spirit of the American Thoracic Society (ATS), European Respiratory Society (ERS), European Society of Clinical Microbiology and Infectious Diseases (ESCMID), and Infectious Diseases Society of America (IDSA) guideline recommendation to document more than one positive culture of the same species to confirm the disease [10]. We differentiated NTM from the *M. tuberculosis* complex using a commercial real-time PCR assay; however, species-level identification and susceptibility testing could not be performed because of resource limitations. The inability to perform MALDI-TOF or sequencing is acknowledged as a major limitation of our report. Nevertheless, repeated isolation from sterile sites, the presence of echocardiographic vegetations and the fatal clinical course in all three cases strongly support true infection rather than contamination.

### Therapeutic considerations

#### Antimicrobial therapy

Evidence-based treatment recommendations for NTM-IE are lacking because most of the experience comes from small case reports and series. The ATS/ERS/ESCMID/IDSA guidelines for NTM pulmonary disease emphasize that treatment should be tailored to the infecting species, extent of disease, drug susceptibility and comorbidities, and that regimens require multiple agents, prolonged courses and careful monitoring for toxicity [10]. Even with appropriate therapy, outcomes remain suboptimal, and reinfection is common [11]. In our series, amikacin and a macrolide (clarithromycin or azithromycin) formed the backbone of therapy, supplemented with linezolid or ceftazidime–avibactam when disease severity or toxicity required. These combinations were empirical and reflected the paucity of standardized regimens; ceftazidime–avibactam was used as salvage therapy in one case of severe sepsis. Drug toxicity was frequent: nephrotoxicity necessitated dose adjustment of amikacin, and thrombocytopenia required the discontinuation of linezolid. These adverse effects underscore the guidelines’ cautioning against prolonged multi-drug therapy [12]. While susceptibility testing would have allowed for more rational regimens, such testing for NTM is often unavailable, especially in resource-limited settings.

#### Surgical management

Surgical removal of infected tissue remains a cornerstone of IE management, especially when caused by virulent or resistant organisms such as *Pseudomonas aeruginosa*. General IE guidelines emphasize that once an indication for surgery exists, such as heart failure, large vegetations, persistent bacteraemia or infection with highly resistant organisms, surgery should not be delayed [13]. Expert opinion advocates ‘operate sooner rather than later’ because damaged valves will not recover and tissue destruction will only progress [13]. In a systematic review of NTM-IE, patients managed without surgery had a much higher mortality rate than those who underwent surgery [2]. Rapidly growing NTM form biofilms that are difficult to eradicate and may require debridement [14]. Despite these principles, surgery was either delayed or not performed in our cases because of diagnostic delay, resource constraints and patient choice. One patient died during induction of valve replacement, and the other two deteriorated before surgery could be arranged. These outcomes highlight the need for early recognition and referral to centres capable of combined medical and surgical management.

#### Association with PCI

Our cases occurred after PCI; however, we cautiously describe the relationship as temporal rather than causal. No procedural breach was documented, and we could not trace a point-source outbreak. Nevertheless, the Centers for Disease Control and Prevention (CDC) notes that NTM can colonize hospital water systems and be disseminated via injections, catheter flushes, hydrotherapy equipment and medical devices [15,16]. The time lag between PCI and the onset of fever (1–5 months) suggests latent contamination, as evident by other healthcare exposures [17]. Therefore, clinicians should consider NTM-IE in any post-procedural patient with persistent fever and negative routine cultures, particularly in settings with limited infection-control infrastructure. In all our three patients, the PCI procedures were uneventful.

#### Outcomes

Recent evidence shows that NTM-IE is a rare but highly lethal disease. In a systematic review of 167 reported cases, 84% of patients had disseminated NTM infection, and the overall mortality approached 45%; mortality was much higher among patients managed medically (66.7%) than among those who underwent combined medical and surgical treatment (30.6%) [2]. Early series of prosthetic valve endocarditis caused by rapidly growing NTM reported mortality rates nearing 88% [18], although more recent case series using aggressive surgical intervention reduced overall mortality to ~23% [18]. These data underscore that invasive NTM infections often require early suspicion, prolonged multi-drug therapy guided by susceptibility testing and timely surgical or device-removal interventions to improve survival [2,18]. All three patients in our series died despite careful medical treatment. High mortality in NTM-IE is consistently reported and may reflect diagnostic delay, advanced disease at presentation, drug toxicity and lack of timely surgery.

This study has several important limitations. First, species-level identification of the NTM isolates could not be performed because advanced diagnostic platforms such as MALDI-TOF mass spectrometry or gene sequencing were unavailable, precluding organism-specific therapeutic optimization. Second, comprehensive molecular sequencing was not performed, which limited phylogenetic characterization and comparison with previously reported strains. Third, the antimicrobial regimens were heterogeneous and individualized, reflecting differences in clinical severity, drug tolerance and resource availability rather than standardized, evidence-based protocols. Fourth, we did not conduct a formal procedural audit or environmental investigation of catheterization laboratory practices to evaluate the possibility of point-source contamination or infection-control breaches. Finally, all cases occurred after recent PCI and provided a temporal rather than causal association; we cannot conclude that the procedures caused the infections. Nevertheless, detailed clinical histories, prolonged culture incubation, repeated isolations and NTM-PCR confirmation using standardized laboratory methodologies can significantly characterize this rare entity.

## Conclusions

This case series highlights the diagnostic complexity and high mortality associated with NTM-IE. Persistent fever after invasive cardiovascular procedures or prolonged, culture-negative endocarditis should prompt consideration of NTM infection. Medical therapy alone is frequently inadequate in NTM-IE, according to most studies, and a combined medical–surgical approach may be required in selected patients, despite inadequate evidence about when and which patients should be operated on.

## Abbreviations

AFB: acid-fast bacilli
AMI: acute myocardial infarction
ATS: American Thoracic Society
BCNE: blood culture-negative infective endocarditis
BIRDEM: Bangladesh Institute of Research and Rehabilitation in Diabetes, Endocrine, and Metabolic Disorders
DAMA: discharged against medical advice
ERS: European Respiratory Society
ESCMID: European Society of Clinical Microbiology and Infectious Diseases
ESR: erythrocyte sedimentation rate
IDSA: Infectious Diseases Society of America
IE: infective endocarditis
NCC: non-coronary cusp
NTM: non-tuberculous mycobacteria
NTM‑IE: non-tuberculous mycobacterial infective endocarditis
PCI: percutaneous coronary intervention
